# In-Built N^+^ Pocket Electrically Doped Tunnel FET With Improved DC and Analog/RF Performance

**DOI:** 10.3390/mi11110960

**Published:** 2020-10-27

**Authors:** Jun Li, Ying Liu, Su-fen Wei, Chan Shan

**Affiliations:** 1 Quanzhou Institute of Equipment Manufacturing, Haixi Institute, Chinese Academy of Sciences, Quanzhou 362216, China; junli@fjirsm.ac.cn; 2 Department of Computer, Quanzhou College of Technology, Quanzhou 362200, China; liuying@foxmail.com; 3 College of Information Engineering, Jimei University, Xiamen 361021, China; weisufen@jmu.edu.cn

**Keywords:** band-to-band tunneling (BTBT), polarity gate (PG), ON-state current, electrically doping, tunnel FETs (TFETs)

## Abstract

In this paper, we present an in-built N^+^ pocket electrically doped tunnel FET (ED-TFET) based on the polarity bias concept that enhances the DC and analog/RF performance. The proposed device begins with a MOSFET like structure (n-p-n) with a control gate (CG) and a polarity gate (PG). The PG is biased at −0.7 V to induce a P^+^ region at the source side, leaving an N^+^ pocket between the source and the channel. This technique yields an N^+^ pocket that is realized in the in-built architecture and removes the need for additional chemical doping. Calibrated 2-D simulations have demonstrated that the introduction of the N^+^ pocket yields a higher *I*_ON_ and a steeper average subthreshold swing when compared to conventional ED-TFET. Further, a local minimum on the conduction band edge (*E*_C_) curve at the tunneling junction is observed, leading to a dramatic reduction in the tunneling width. As a result, the in-built N^+^ pocket ED-TFET significantly improves the DC and analog/RF figure-of-merits and, hence, can serve as a better candidate for low-power applications.

## 1. Introduction

A tunnel field-effect transistor (TFET) is considered to be one of the most promising candidates for low-power applications [1,2,3,4,5]. TFET devises transport carriers by band-to-band tunneling (BTBT), which differs from the drift-diffusion working principle of conventional metal-oxide-semiconductor field-effect transistor (MOSFET). Theoretically, TFET can break through the limit of thermoelectric potential, obtain an ultra-steep subthreshold swing (SS) below 60 mV/dec at room temperature, and achieve extremely high on/off current ratio at very low voltage. However, the low ON-state current of TFET, originating from the large tunneling resistance at the tunneling junction, limits its application in high-speed integrated circuits [6]. Source-pocket (PNPN) TFETs have been investigated to overcome these shortcomings [7,8]. The PNPN TFET has the same structure as the conventional p-i-n TFET, except that a narrow N^+^ doped pocket is introduced between the source and the channel. Compared with the conventional TFET, the PNPN TFET exhibits an increased ON-state current, enhanced SS, and improved device reliability [9,10]. Although the introduction of the N^+^ pocket can improve electrical characteristics, it is still a technical challenge to realize such a narrow and highly doped pocket [11,12,13,14].

To address the aforementioned issues, we propose an in-built N^+^ pocket electrically doped TFET (ED-TFET) using the polarity bias concept [15,16,17], where the narrow N^+^ pocket is realized in the in-built architecture and does not require additional chemical doping. Recently, an ED-TFET with bandgap engineering for analog/RF applications has been reported, which uses the polarity bias concept on a junctionless (JL) N^+^ starting structure and shows simplicity in fabrication steps [18]. However, using calibrated two-dimensional simulations, we demonstrate that the proposed in-built N^+^ pocket ED-TFET exhibits improved DC and analog/RF characteristics compared to the conventional Silicon-based ED-TFET.

In this work, we investigate device design and DC and analog/RF performances of the proposed transistors with regard to several key parameters. First, the device concept, as well as the principle of operation, is discussed in Section 2. Second, simulation results and considerations for optimal design are described in Section 3. Finally, Section 4 draws conclusions by summarizing the attractive properties of the proposed in-built N^+^ pocket ED-TFETs.

## 2. Device Structure and Operating Principle 

**Figure 1:**
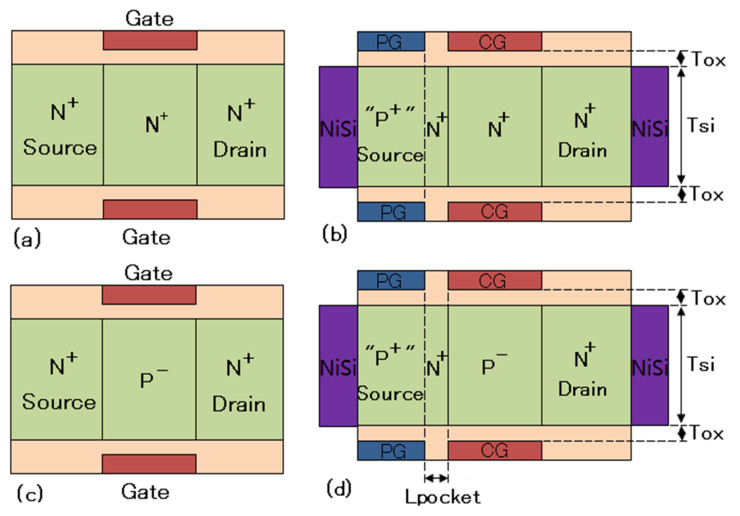
Cross-sectional views of (**a**) Beginning JL FET structure to realize conventional electrically doped TFET (ED-TFET), (**b**) Conventional ED-TFET [19], (**c**) Beginning MOSFET structure to realize the in-built N^+^ pocket ED-TFET, (**d**) In-built N^+^ pocket ED-TFET. JL FET, junctionless field-effect transistor; ED-TFET, electrically doped tunnel field-effect transistor; MOSFET, metal-oxide-semiconductor field-effect transistor.

To investigate the DC and analog/RF performance of an ED-TFET with an insertion of an N^+^ pocket, the proposed device has been simulated in comparison with a conventional ED-TFET without an N^+^ pocket. Figure 1a,b show the cross-sectional views of the starting JL field-effect transistor (FET) structure and the final structure of conventional ED-TFET, respectively. Figure 1c,d show the cross-sectional views of the starting MOSFET structure and the final structure of the proposed in-built N^+^ pocket ED-TFET. Both types of devices are composed of two sets of gate electrodes: a control gate (CG) and a polarity gate (PG). The effective tunneling barrier of the devices can be modulated by adjusting the work function of the CG. In addition, the PG embedded on the source side is used to convert part of the N^+^ doped source into a “P^+^” region, as depicted in Figure 1b,d. To create a P^+^ source region, the PG terminal is biased by an adequate negative voltage to increase the carrier concentration to ~10^19^ cm^−3^. Source and drain contacts are composed of nickel silicide (NiSi) with a Schottky barrier height of 0.45 eV. 

The main fabrication processes of the proposed in-built N^+^ pocket ED-TFET are listed as in the following steps. In the first step, the devices can be fabricated using nonplanar technologies, exploiting deep reactive ion etching (DRIE) or Bosch processes to form device channels [18]. The gate oxide is then deposited on the whole channel following by the deposition of the control gate. After that, the polarity gate is patterned all around the channel using e-beam lithography. After both sets of gates are formed, the spacers are patterned, and a nickel layer is deposited to generate NiSi at the source and drain contacts [16]. 

The major difference between conventional ED-TFET and the proposed in-built N^+^ pocket ED-TFET lies in the doping concentration in their starting structures. In a conventional ED-TFET, the film is uniformly doped with no p-n junctions. Using the concept of polarity bias, the N^+^-N^+^-N^+^ film (source, channel, and drain) is converted into P^+^-I-N^+^ gated structure, similar to a conventional TFET. The spacer thickness between the CG and PG is chosen to be 5 nm to obtain the optimum simulation results in ED-TFET [19]. In order to realize the in-built N^+^ pocket ED-TFET, it is necessary to construct a MOSFET as a beginning structure in which the source, channel, and drain are N^+^, P^-^, N^+^ doped, respectively, as shown in Figure 1c. The role of the polarity gate is to form a P^+^ region at the source. This creates the N^+^ doped pocket between the source and the channel, thereby achieving the same doping sequence as in the PNPN TFET, as illustrated in Figure 1d. This method realizes the formation of a narrow N^+^ pocket in the ED-TFET without the additional chemical doping process. The device design parameters that we have used in simulations are listed in Table 1. In order to have an N^+^ pocket in the proposed ED-TFET, the channel doping switches from an n-type with a concentration of 1 × 10^19^ cm^−3^ to a p-type with a concentration of 1 × 10^17^ cm^−3^. Furthermore, to reduce the ambipolar current, a drain doping of N_D_ = 5 × 10^18^ cm^−3^ is used. The N^+^ pocket length (*L*_pocket_) varies from 1 nm to 9 nm to maintain the expected tunneling performance. Making the layer underneath the polarity gate intrinsic, the work functions of the PG in conventional and proposed ED-TFET are chosen to be 4.33 eV and 4.74 eV, respectively. The simulation technique requires the source terminals to be grounded (*V*_S_ = 0). Hence, we have considered *V*_CG_ = *V*_CGS_ and *V*_PG_ = *V*_PGS_ in all the devices.

**Figure 2:**
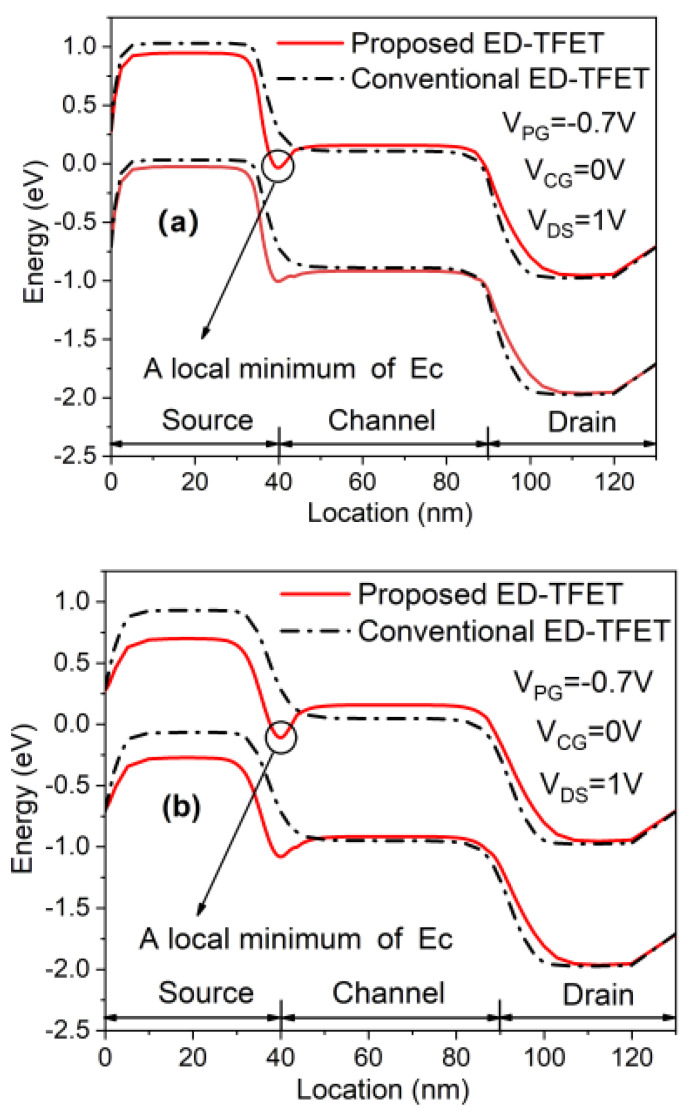
Energy band diagrams at (**a**) 1 nm and (**b**) 5 nm below the Si-oxide interface of the conventional and proposed ED-TFET at OFF-state.

The working mechanism of the proposed ED-TFET is consistent with that of a conventional PNPN TFET. Figure 2 shows the energy band diagrams for the proposed and conventional ED-TFET at 1 nm and 5 nm below the Si-oxide interface. A local minimum point appears on the conduction energy band edge (*E*_C_) at *V*_CG_ = 0 V. This happens because the introduction of the N^+^ pocket leads to a lowering of the *E*_C_ curve and a rapid reduction in the tunneling barrier width due to the alignment of this local minimum with the valence energy band edge (*E*_V_) at the source [20,21,22]. Conventional ED-TFET does not have this local minimum on the *E*_C_ curve, as illustrated in Figure 2. Overall, tunneling efficiency is expected to be improved due to the reduced tunneling barrier width as compared to conventional ED-TFETs.

## 3. Simulation Results and Discussions

This section describes the simulated DC and analog/RF performance of the proposed device in ultra-low power applications. First, the DC transfer characteristics of the proposed and conventional ED-TFETs are simulated. Then, the influence of the critical structural parameters on the performance of the proposed device is discussed. Finally, using AC signal analysis, the enhancement in the analog/RF performance of the proposed ED-TFET is evaluated through the comparison to its conventional counterpart. 

### 3.1. DC Characteristics

All ED-TFET structures are simulated using the Silvaco Atlas device simulation tool [23]. In reality, band-to-band tunneling (BTBT) is a nonlocal process, and the spatial variation of the energy bands should be accounted for. Therefore, a non-local BTBT model is chosen prior to a local BTBT to consider the tunneling along the lateral direction for both devices. Besides, the Lombardi mobility model is included to take into account mobility degradation owing to the electric field. In addition, the concentration-dependent Shockley–Read–Hall (SRH) recombination model is applied and combined with the Auger recombination model. Bandgap narrowing (BGN) model is enabled to account for the highly doped regions of both devices. Besides, Fermi–Dirac statistics is incorporated in the simulation [24]. An HfO_2_ gate dielectric with a physical thickness of approximately 4.5 nm and an equivalent oxide thickness (*EOT*) of 0.8 nm is used in the simulations. The quantum confinement (QC) model is not included. The direct tunneling model is not utilized because of the assumption of high-κ/metal gate-stack technology [25]. Simulation models are verified by reproducing the results reported in [26]. 

**Figure 3:**
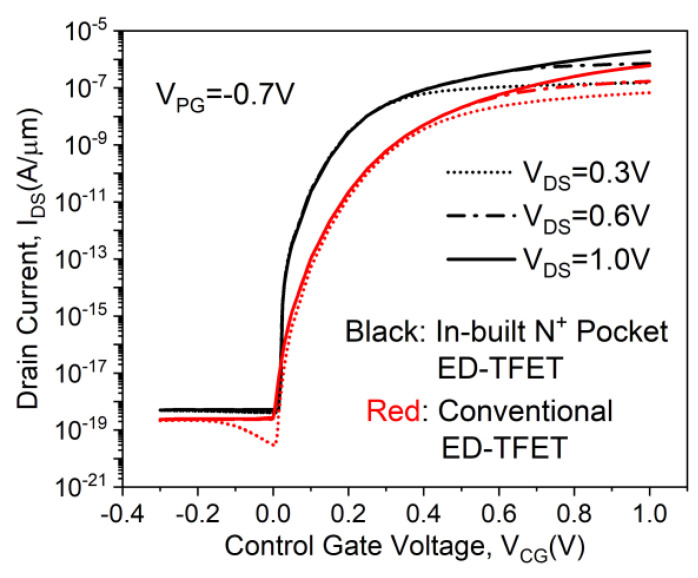
Transfer characteristics of conventional and in-built N^+^ pocket ED-TFET for different *V*_DS_.

The transfer characteristics of the conventional ED-TFET and the proposed in-built N^+^ pocket ED-TFET under different *V*_DS_ biases from 0.3 to 1V are compared in Figure 3. The length of the N^+^ pocket (*L*_pocket_) is fixed at 5 nm. For fair comparisons, all simulations use a control gate work-function that achieves *V*_onset_ = 0 V for both devices. The value of the control gate work-function is ~4.37 eV in the proposed device, which can be achieved by using metal gates. We observe from Figure 3 that the ON-state current (*I*_ON_) and the subthreshold slope (SS) are significantly improved in the proposed device. This happens due to the formation of the local minimum point in the conduction energy band edge discussed above. Noted that the displayed SS is underestimated in the absence of the QC model. It can be inferred that the ON-state current of the proposed ED-TFET has improved by a factor of 3 over the conventional ED-TFET without the N^+^ pocket. The *I*_ON_ is regarded as a drain current (*I*_DS_) at *V*_CG_ = *V*_DS_ = 1 V. For the proposed device and the conventional ED-TFET, the average SS extracted according to the literature [27] is 28 mV/decade and 57 mV/decade, respectively. Moreover, the ON-state current of the proposed ED-TFET can be further enhanced by using low bandgap materials, heterostructures, and strain technology.

### 3.2. Device Optimizations

**Figure 4:**
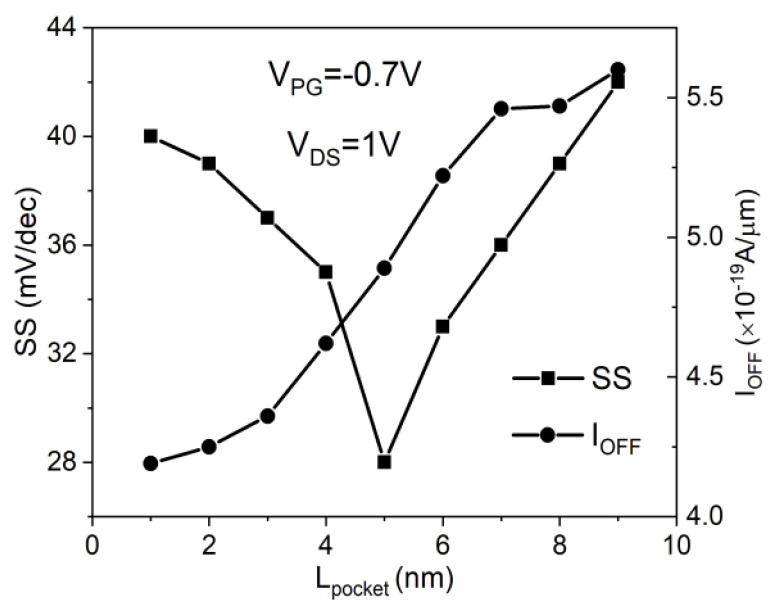
Average subthreshold swing (SS) and OFF-state current (*I*_OFF_) of the in-built N^+^ pocket ED-TFET as a function of the length of the pocket (*L*_pocket_).

**Figure 5:**
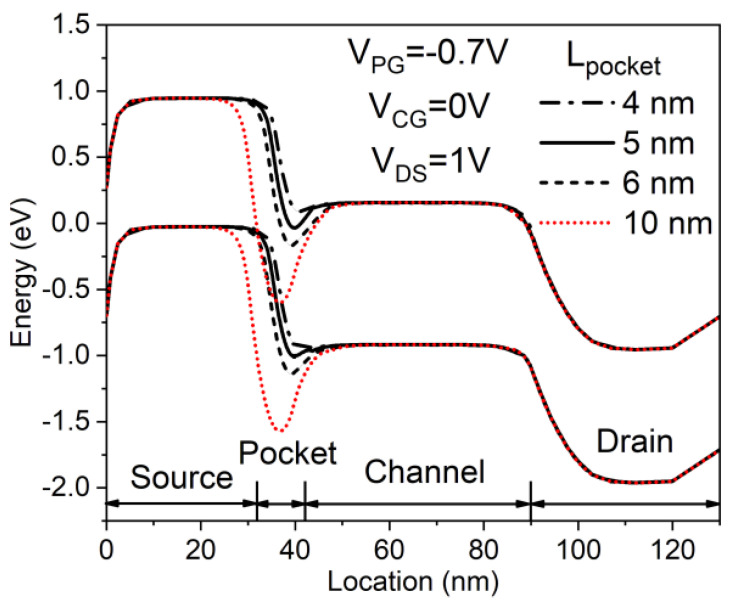
OFF-state energy band diagram for the in-built N^+^ pocket ED-TFET for different pocket lengths (*L*_pocket_).

Optimizing the design of the in-built N^+^ pocket ED-TFETs is achieved by optimizing *L*_pocket_. Figure 4 depicts SS and *I*_OFF_ as a function of *L*_pocket_. To find the optimum length of N^+^ pocket, *L*_pocket_ is adjusted in the range of 1 to 10 nm. The *I*_OFF_ is defined as a drain current (*I*_DS_) at *V*_CG_ = 0 V and *V*_DS_ = 1 V. Figure 5 illustrates the energy band diagram of the in-built N^+^ pocket ED-TFETs with varying *L*_pocket_ values at OFF-states (*V*_CG_ = 0 V). In the case of *L*_pocket_ = 4 nm, the depth of the conduction band well where the local minimum of *E*_C_ is located is significantly reduced, making it difficult to induce band-to-band tunneling. This degrades the SS as *L*_pocket_ drops below 4 nm. However, when *L*_pocket_ exceeds 6 nm, the width of the conduction band well increases, resulting in fewer abrupt transitions between ON- and OFF-states. As a result, the subthreshold characteristics significantly deteriorate. In addition, as *L*_pocket_ is increased to 10 nm, an energy barrier forms between the N^+^ pocket and the channel, similar to an n-channel MOSFET. Under this condition, the carrier injection mechanism switches from band-to-band tunneling to diffusion over the barrier. Taking all these effects into account, the optimal length of the N^+^ pocket is found to be 5 nm. 

**Figure 6:**
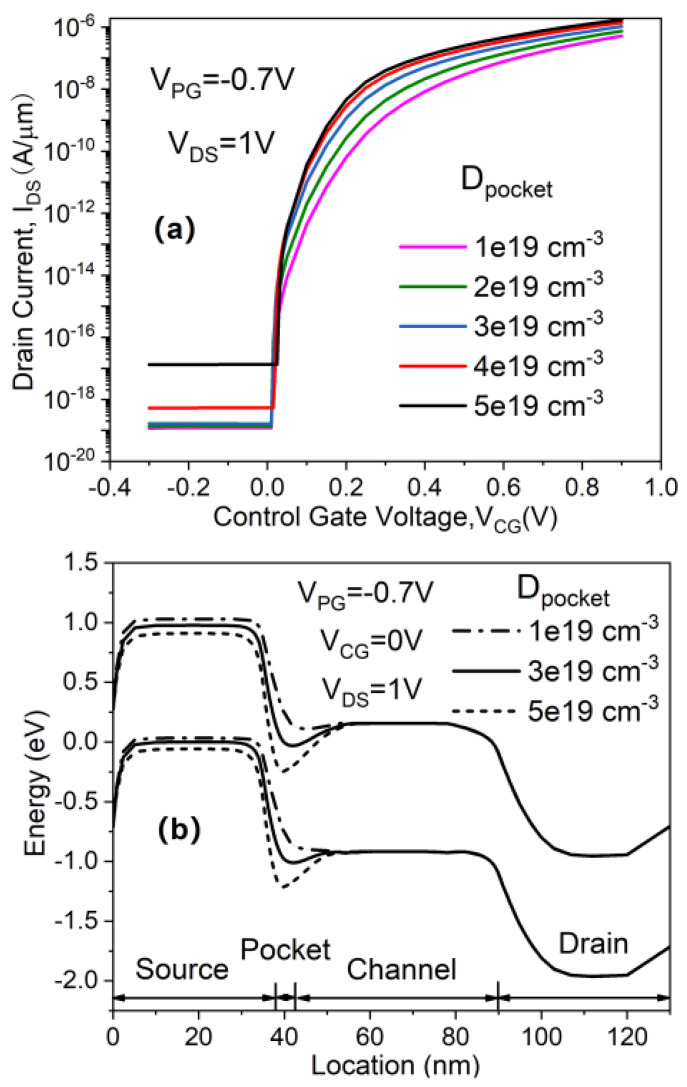
Impact of *D*_pocket_ on (**a**) transfer characteristics and (**b**) OFF-state energy band diagram of the in-built N^+^ pocket ED-TFET.

A laterally modulated energy band is obtained with an N^+^ pocket inserted in the proposed device. The doping concentration of the N^+^ pocket (*D*_pocket_), therefore, has an important influence in determining the improvement of electrical characteristics. Figure 6a,b illustrate the impact of *D*_pocket_ on the transfer characteristics and the OFF-state band diagrams of the in-built N^+^ pocket ED-TFET. For the optimized device with *L*_pocket_ = 5 nm, both ON-state current and SS are enhanced with an increase in *D*_pocket_, as depicted in Figure 6a. The reason can be inferred from Figure 6b that as *D*_pocket_ decreases to 1 × 10^19^ cm^−3^, the depth of the conduction band well in which the local minimum of *E*_C_ is located significantly reduces, thus leading to a reduced *I*_ON_ and a degraded SS. As *D*_pocket_ is increased from 1 × 10^19^ cm^−3^ to 5 × 10^19^ cm^−3^, *I*_OFF_ dramatically increases, although *I*_ON_ only increases slightly, as shown in Figure 6a. The N^+^ pocket becomes partially depleted at higher *D*_pocket_ values, resulting in an increased *I*_OFF_. From this point of view, 4 × 10^19^ cm^−3^ is chosen as the optimal value of *D*_pocket_.

**Figure 7:**
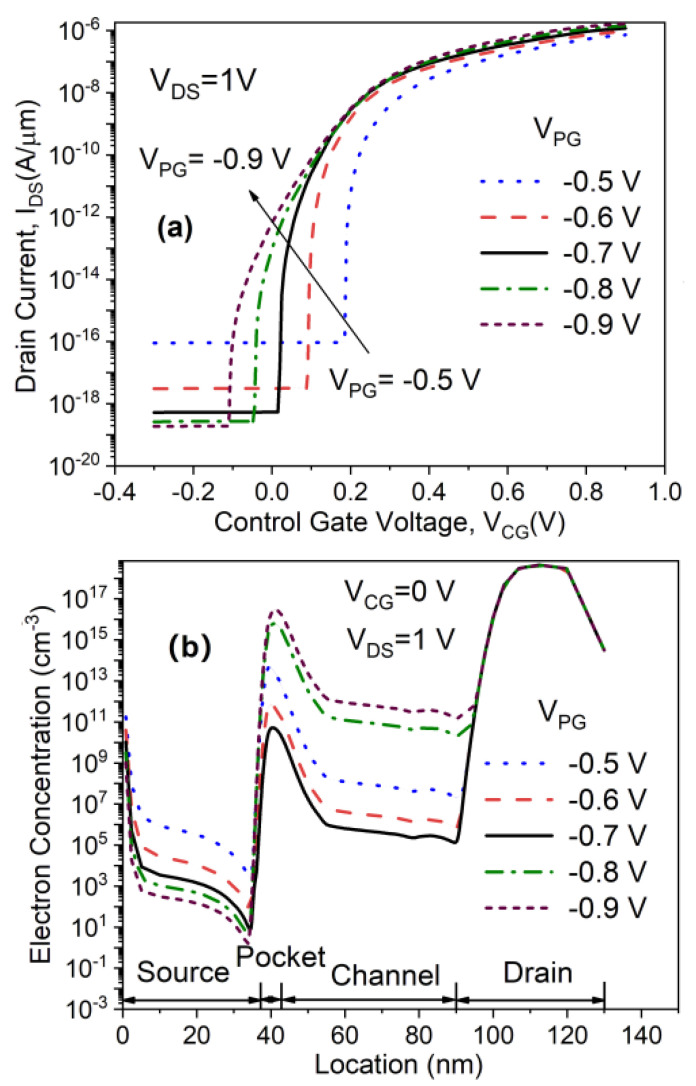
Impact of polarity gate (PG) bias on (**a**) transfer characteristics and (**b**) electron concentration of the in-built N^+^ pocket ED-TFET.

In the proposed device, an adequate negative bias is applied at the PG terminal to generate a P^+^ source region with a hole concentration similar to its conventional counterpart, as mentioned above. Therefore, the PG terminal bias (*V*_PG_) needs to be optimized as well. Figure 7a,b show the transfer characteristics and electron concentration of the in-built N^+^ pocket ED-TFET as a function of *V*_PG_. One can observe from Figure 7a that the scaling of |*V*_PG_| causes an increase in the OFF-state current, which is consistent with the previously reported results [17]. Furthermore, the OFF-state leakage current is drastically increased when *V*_PG_ = −0.5V. However, the ON-state current is slightly reduced as |*V*_PG_| is scaled. The decrease in |*V*_PG_| results in a lower vertical electric field, which, in turn, leads to a decrease in the number of holes. Thus, an increase in |*V*_PG_| causes a reduction in electron concentration in the source region of the in-built N^+^ pocket ED-TFET, as shown in Figure 7b. In addition, it can be seen from Figure 7a that the optimal PG bias in terms of average SS is −0.7 V. This can be understood from the electron concentration distribution with different PG bias at OFF-state. In the case of the proposed device with *V*_PG_ = −0.7 V, the electron concentration in the pocket region shows the lowest value, as illustrated in Figure 7b. When *V*_PG_ increases above or decreases below this value, the N^+^ pocket begins to be partially depleted at OFF-state, and the electron concentration in the pocket region increases. This increased electron concentration in the partially depleted N^+^ pocket affects the conduction band profile at OFF-state. As a result, the conduction band well becomes wider, resulting in SS optimum value for the PG bias of the in-built N^+^ pocket ED-TFET with the lowest SS and a considerably high *I*_ON_/*I*_OFF_ ratio (~10^12^).

### 3.3. Analog/RF Performance

**Figure 8:**
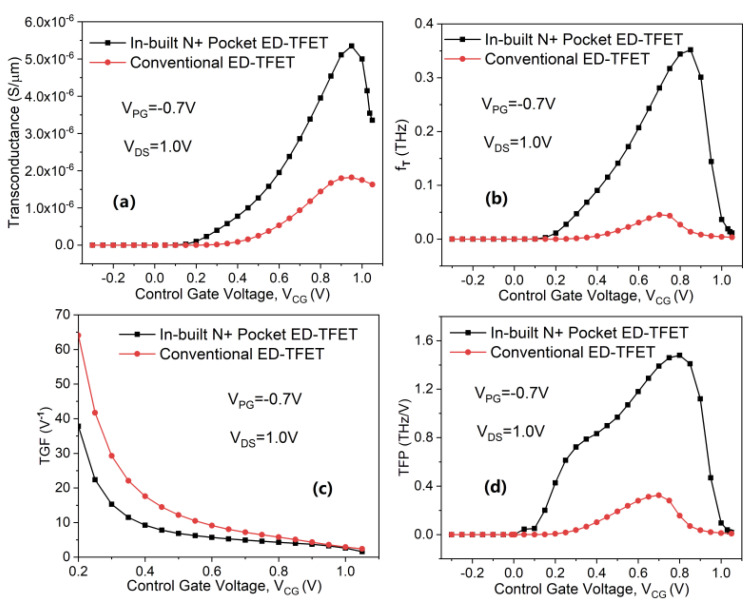
Variation of (**a**) transconductance, (**b**) cutoff frequency, (**c**) TGF, and (**d**) TFP along *V*_CG_ of the conventional and in-built N^+^ pocket ED-TFET.

The analog/RF performance of the in-built N^+^ pocket ED-TFET is simulated and compared with a conventional ED-TFET having identical physical dimensions. Therefore, the analog/RF figure-of-merits (FOMs) are investigated, including transconductance (*g_m_*), transconductance-to-drain current ratio, also known as transconductance generation factor (TGF), cutoff frequency (*f_T_*), and transconductance frequency product (TFP). Transconductance is considered a critical parameter for obtaining high gain and *f_T_* in analog circuit applications [28,29]. The *g_m_* of the device is calculated by the slope of the log(*I*_DS_)–*V*_CG_ curve when *V*_DS_ remains constant, which can be expressed as *g_m_* = d*I*_DS_/d*V*_CG_. Figure 8a compares the *g_m_* characteristics of a conventional and the proposed ED-TFETs as a function of *V*_CG_. It can be seen that the *g_m_* of the proposed in-built N^+^ pocket ED-TFET is larger than that of the conventional ED-TFET. For the proposed structure, *I*_DS_ changes greatly with *V*_CG_, while *I*_ON_ maintains a high value, resulting in a higher *g_m_*. In addition, it can be inferred that *g_m_* increases with the increase of *V*_CG_ until it enters the saturation region. The increase of the BTBT generation rate directly leads to an increase in *g_m_*. However, it decreases at higher *V*_CG_ due to reduced mobility.

For RF applications, the cutoff frequency (*f_T_*) is another important parameter. This is defined as the frequency at which the short circuit current gain reaches unity and can be expressed as *f_T_* = *g_m_* /2π(*C*_gs_ + *C*_gd_). Generally, *f_T_* should be as high as possible to enable the device to be used broadly in high-frequency circuit applications. Figure 8b shows the dependence of *f_T_* on *V*_CG_. It can be inferred that the significant improvement in *f_T_* of the in-built N^+^ pocket ED-TFET is due to its larger *g_m_* compared to the conventional counterpart. It can be clearly seen from the figure that this rapid increase in *g_m_* results in an increase in *f_T_* until *V*_CG_ reaches 0.8 V. After that, a sharp drop in *g_m_* and an increase in gate capacitance results in a decrease in the *f_T_*. The proposed and conventional ED-TFETs achieve a maximum *f_T_* of 0.352 and 0.045 THz, respectively.

TGF is another critical parameter that quantifies the device efficiency [30] and can be expressed as TGF = *g_m_*/*I*_DS_. The variation in TGF with *V*_CG_ for both ED-TFETs is shown in Figure 8c. The proposed device has a lower TGF compared to its conventional counterpart. This is happening because, in the case of TGF, the drain current is dominant as compared to *g_m_*. When the control gate voltage is high, the drain current increases rapidly, resulting in a corresponding decrease in the TGF. The TFP is another key FOM for high-frequency circuits and is essentially the product of the TGF and *f_T_*, which is expressed as TFP = (*g_m_*/*I*_DS_) × *f_T_* [31]. From Figure 8d, it can be observed that the proposed ED-TFET has a higher value of TFP compared with the conventional ED-TFET. The improvement in TFP is due to the higher *f_T_*. This simulation analysis shows that overall the in-built N^+^ pocket ED-TFET appears to be more suitable for RF applications than conventional ED-TFETs.

## 4. Conclusions

In this paper, we have presented a method to insert an N^+^ pocket in an ED-TFET by using the polarity bias concept. This N^+^ pocket is realized without the need for additional chemical doping. In addition, device design has been optimized by modulating *L*_pocket_, *D*_pocket_, and *V*_PG_. The DC and analog/RF performance is evaluated using 2-D simulations. At the optimized dimensions, the in-built N^+^ pocket ED-TFET has a better simulated performance to the conventional ED-TFET in terms of SS, *I*_ON_, *g_m_*, *f_T_*, TGF, and TFP. The enhancement in SS and *I*_ON_ is attributed to a local minimum of *E*_C_, which is formed by the introduction of the N^+^ pocket, resulting in a higher *g_m_* and thereby an increase in *f_T_* and TFP. The in-built N^+^ pocket ED-TFET appears to be an attractive candidate for future low power applications.

## Figures and Tables

**Table 1:** Parameters used for device simulation.

Parameter	Conventional ED-TFET ^1^	In-Built N+ pocket ED-TFET
Effective Gate Oxide Thickness (*EOT* ^2^)	0.8 nm	0.8 nm
Silicon Film Thickness (*T*_Si_)	10 nm	10 nm
Control Gate Length	50 nm	50 nm
Spacer Thickness between CG ^3^ and PG ^4^	5 nm	1~9 nm
Channel Doping	1 × 10^19^ cm^−3^ (N^+^)	1 × 10^17^ cm^−3^ (P^-^)
Source Doping	1 × 10^19^ cm^−3^ (N^+^)	4 × 10^19^ cm^−3^ (N^+^)
Drain Doping	1 × 10^19^ cm^−3^ (N^+^)	5 × 10^1^^8^ cm^−3^ (N^+^)
Control Gate Work-Function	4.74 eV	4.74 eV
Polarity Gate Work-Function	4.74 eV	4.33 eV

^1^ ED-TFET: electrically doped tunnel FET; ^2^ *EOT*: equivalent oxide thickness; ^3^ CG: control gate; ^4^ PG: polarity gate.
